# Cloning, Expression and Purification of *Pseudomonas putida ATCC12633* Creatinase

**Published:** 2017

**Authors:** Elnaz Afshari, Zahra Amini-bayat, Saman Hosseinkhani, Nahid Bakhtiari

**Affiliations:** 1. Department of Microbiology, Islamic Azad University of Pharmaceutical Sciences Branch, Tehran, Iran; 2. Department of Biotechnology, Iranian Research Organization for Science and Technology (IROST), Tehran, Iran; 3. Department of Biochemistry, Faculty of Biological Sciences, Tarbiat Modares University, Tehran, Iran

**Keywords:** Creatine, Creatinase, *Pseudomonas putida*

## Abstract

**Background:**

*Pseudomonas putida (P. putida)* ATCC12633 can produce creatinase. It is a microbial enzyme which degrades creatinine in bacteria and provides source of carbon and nitrogen. Also, this enzyme is used in the enzymatic measurement of creatinine concentration for diagnosis of renal and muscles functions and diseases. Our purpose was recombinant production of creatinase for using in clinical measurement of serum or urine creatinine.

**Methods:**

A 1209bp of open reading frame of creatinase was amplified by PCR from *P. putida ATCC12633* genome and cloned into pET28a expression vector which was digested using NheI and XhoI restriction enzymes. Cloning was confirmed by colony PCR, double digestion analysis and sequencing. Recombinant pET28a vector was transformed to *Escherichia coli (E. coli) BL21 (DE3)*. Creatinase expression was induced in *E.coli BL21 (DE3)* using IPTG and confirmed by SDS-PAGE and western blotting. Purification of creatinase was performed using Ni-NTA column. The specific activity of this enzyme was also investigated.

**Results:**

The creatinase gene cloning was confirmed by DNA sequencing. Successful expression of creatinase was performed in *E. coli* (57.4% of total protein). SDS-PAGE and western blot analysis showed a 45 *kDa* creatinase protein. Purification of creatinase was done with high purity. The specific activity of recombinant enzyme is 26.54 *unit/mg* that is much higher than other creatinase used in the commercial kits (9 *unit/mg*).

**Conclusion:**

The *P. putida ATCC12633* recombinant creatinase was expressed efficiently in *E. coli BL21* and 57% of total protein was the recombinant creatinase. Also, expressed creatinase has high solubility and also the enzyme has good activity compared to enzymes used in commercial kits, so a new source of creatinase was produced for creatinine assay kit in this study.

## Introduction

Creatinase [creatine amidinohydrolase (EC 3.5.3.3)] converts creatine to sarcosine and urea ^1–3^. This enzyme is used for creatine and creatinine assessment in biological fluids ^3^. Creatine and creatinine levels are the most important diagnostic markers in the assay of RBC age ^4–7^, thyroid, renal and muscular functions ^8,9^ and in the diagnosis of anemia ^10,11^. In human body, creatine is naturally produced in kidney, liver and pancreas from aminoacids such as L-arginine, glycine and L-methionine ^12^, then creatine can be transported to muscles, brain and heart tissues through the blood-stream. It is phosphorylated to phosphocreatine by creatine kinase and stored as the source of energy. During the muscle contraction, phosphocreatine is converted to creatinine. Finally, creatinine is absorbed by kidney and is especially excreted through urine ^13^. So the metabolism of creatine has been studied as a relationship between creatine uptake and creatinine excretion ^14^.

Disturbance of creatine metabolism is shown by reduced concentration of muscle creatine, decreased excretion of urine creatinine and increased excretion of urine creatine in muscular dystrophy patients ^15,16^. Also, increased concentration of creatinine is shown by reduced glomerular filtration rate in renal diseases. Creatine and creatine phosphate are changed into creatinine nonenzymatically in vertebrates but there are a growing number of microorganisms that express specific enzymes for the degradation of creatinine. One of these enzymes is creatinase, an inducible enzyme expressed in bacteria when creatinine is provided as the main source of carbon or nitrogen ^17–22^. The creatine decomposing enzyme (creatinase) with 403 amino acids is a homodimeric enzyme (with small N-terminal domain and large C-terminal domain) that is classified as a member of the hydrolases family of enzymes ^3,23^. This enzyme was first identified in two species of *Pseudomonas* (*P. eisenbergii* and *ovalis*) ^20^. The other bacteria which produce creatinase are as follows: *Pseudomonas* ^2,24^, *Bacillus* ^25^, *Flavobacterium* ^1^, *Micrococcus* ^26^, *Alcaligenes*, *Clostridium* ^27^, *Athrobacter* ^28^, and *Paracoccus* ^9^. The routine technique for creatinine measurement is the Jaffe reaction that is non-specific and many substances could interfere in this assay. Due to lack of interference and being specific, the enzymatic methods are preferred. One of the enzymatic methods is as follows: at first creatinine is converted to creatine by creatininase (EC 3.5.2.10), then creatine is hydrolyzed to urea and sarcosine by creatinase (EC 3.5.3.3). In the third step, sarcosine is demethylated to H_2_O_2_ by sarcosine oxidase (EC 1.5. 3.1) ^3^. Finally, H_2_O_2_ can be measured by spectrophotometric methods.

Extraction of enzymes from natural sources is hard and it does not meet the requirement for quantity. Recombinant technology using different expression hosts is the method of choice among all recombinant expression hosts and *Escherichia coli* (*E. coli)* is the most conventional organism.

Because creatinase is an enzyme that is used for clinical application, its activity is an important property. To the best of our knowledge, the creatinase that has been used in commercial kits has maximum activity of 9 *unit/mg*, so availability of enzyme with higher activity is distinctly a need. On the other hand, in industrial production, increasing expression yield is desirable.

The aim of this study was to investigate if ATCC-12633 creatinase has better activity than commonly used commercially creatinases. So, *Pseudomonas putida (P. putida)* ATCC12633 creatinase gene was amplified from this strain genome and cloned into pET28a expression vector. The recombinant DNA was expressed in *E. coli BL21 (DE3)* and verified by western blot analysis then purified by affinity chromatography and its activity has been determined.

## Materials and Methods

### Materials and bacterial strains and vector

*P. putida* ATCC12633 was used as the source of creatinase gene (Persian Type Culture Collection (IRO-ST PTCC, Iran)). *E. coli DH5α* strain (Novagen, Germany) and *E. coli BL21 (DE3)* were used as cloning and expression hosts and the pET28a(+) vector (Novagen, Germany) was used as expression vector. Kanamycin was obtained from duchefa, Taq polymerase, 2x PCR master mixes, 1 *kb* DNA Ladder, NheI and XhoI Restriction Enzymes, T4 DNA Ligase, IPTG (isopropyl-β-D-thiogalactopyranoside) and Protein ladder were obtained from fermentas. Ni-NTA agarose resin was from QIAGEN and Anti poly-Histidine Antibody conjugated with HRP was from Sigma. Creatine, DMSO (Dimethylsulfoxide), DMAB (4-Dimethyl aminobenzaldehyde), and Imidazole were obtained from Merck. YTA DNA extraction, plasmid extraction and gel extraction kits (Yekta Tajhiz Azma, Iran) were used in this study as well.

### Genomic DNA extraction and amplification of creatinase gene

Genome of *P. putida* ATCC12633 has been sequenced in 2014 ^29^ and according to the blast analysis, one of its gene was predicted as a gene encoding creatinase. On the other hand, in our lab, the bacteria showed creatinase activity in the presence of creatinine as the unique organic source.

The genomic DNA of *P. putida* was extracted using the YTA DNA extraction mini kit (Yekta Tajhiz Azma, Iran). Based on creatinase nucleotide sequence which was obtained from NCBI database (accession number: NC_021505.1), two specific oligonucleotide primers were designed by oligoanalyzer software and were synthesized by Bioneer company. The complete open reading frame of creatinase gene was amplified using forward primer: 5′-CTATA**GCTAGC**CAAATGCCCA AGACCCTG-3′ which contained a NheI restriction site and reverse primer: 5′-GGC**CTCGAG**TTATTTGCGA ATGATGTTGTG-3′ which contained a XhoI restriction site (underlined). Amplification was carried out by thermocycler (Palm Cycler hp ipaq) under the following program: denaturation at 95°*C* for 3 *min* followed by 30 cycles at 95°*C* for 30 *s*, 63°*C* for 30 *s*, and 72°*C* for 80 *s* and a 10 *min*-final extension at 72°*C*. To assess the quality and quantity of amplified gene, 0.7% agarose gel electrophoresis was used. YTA gel extraction kit was used to purify PCR products.

### Cloning of creatinase gene into expression vector

pET28a(+) was extracted using YTA plasmid DNA extraction kit. PCR product and plasmid were double digested with NheI and XhoI restriction enzymes and gel purified using YTA gel extraction kit, then ligation was performed at 3:1 vector/insert ratio. Preparation of *E. coli DH5α* competent cells was performed by FB buffer method ^30^. Then, recombinant plasmid was transformed to *E. coli DH5α* by chemical method. Colony PCR, double digestion and nucleotide sequencing were performed to confirm gene cloning. Colony PCR was done using T7 terminator and T7 promoter primers under the following program: denaturation at 94°*C* for 5 *min* followed by 30 cycles at 94°*C* for 60 *s*, 50°*C* for 90 *s*, and 72°*C* for 90 *s* and a 10 *min*-final extension at 72°*C*. PCR products were analyzed using 1% agarose gel electrophoresis.

### Expression and solubility analysis by SDS-PAGE

The recombinant pET28a-cre vector was transformed into *E. coli BL21(DE3)* competent cells. The recombinant creatinase was expressed as N-terminal fusion to His-tag. The creatinase expression was done in LB broth medium containing kanamycin (50 *μg/ml*) at 37°*C* with 1 *mM* IPTG for two different times, 4 *hr* and overnight. The same method was used for negative control (a colony containing pET28a without cre gene). The cells were harvested at 4*°C* and 8000 *rpm* for 20 *min*. For disrupting the cells, pellet was resuspended in lysis buffer (containing: 10 *mM* Imidazole, 300 *mM* NaCl, 50 *mM* NaH_2_PO_4_ in pH=8) then different methods such as grinding with liquid nitrogen and sonication (Misonix 600W) with amplitude of 20%, 10 *s* ON, 20 *s* OFF were used. Finally, cell lysate was analyzed for total (containing: soluble and insoluble proteins) and soluble recombinant protein by 12% SDS-PAGE.

### Plasmid stability test

One of the most important problems in the production of recombinant proteins is the tendency of transformed cells to lose their engineered properties following loss of their plasmids. Instability of plasmids can result in a significant loss in productivity ^31^. For stability test, the serial dilutions (10^−1^, 10^−2^, 10^−3^, 10^−4^, 10^−5^) of transformed cells were prepared, then 50 *μl* of diluted samples were plated on selective (containing kanamycin) and nonselective (without kanamycin) LB plates and let to grow for 16/18 *hr* in 37*°C*.

### Western blot analysis

Soluble and total fractions of bacterial lysate were subjected to electrophoresis under reduced conditions using a 12% SDS-PAGE gel and transformed to a nitrocellulose membrane by Bio-rad transfer system (Schleicher & Schuell). Then, membrane was blocked by 5% skim milk in TBS-T and immunoblotted with anti his-tag antibody conjugated to HRP at a 1:2000 dilution. Protein bands were developed using DAB (3, 3′-diaminobenzidine) and H_2_O_2_ in PBS containing NiCl_2_ and kept in a dark place for appearance of the protein band.

### Protein purification

For purification by native method, transformed cell harboring pET28-cre plasmid was grown at a large scale under the following conditions: in LB broth, OD_600_ *_nm_*=1, 1 *mM* IPTG at 37*°C* for 4 *hr*. Then, cells were harvested by centrifugation for 20 *min* at 4*°C* at 8000 *rpm* and resuspended in 15 *ml* lysis buffer (containing 10 *mM* imidazole) and stored for overnight at −20*°C*. Cell pellets were sonicated for 10 *min* and centrifuged at 8000 *rpm* at 4*°C* for 20 *min*. Supernatant that included soluble form of recombinant creatinase was added to Ni-NTA column and column was washed with wash buffer (containing 20 *mM* imidazole) and finally recombinant protein was eluted using elution buffer (containing 250 *mM* imidazole) (Ni-NTA Aga-rose; Qiagen). The purity of fractions was analyzed by 12% SDS-PAGE.

### Recombinant creatinase concentration and activity assay

The creatinase concentration was obtained using Bradford method ^32^ and creatinase activity was measured by estimating the amount of urea formation from the hydrolysis of creatine in a colorimetric assay. For this reason, 0.1 *ml* of recombinant creatinase solution was added to 0.9 *ml* of 50 *mM* phosphate buffer containing 100 *mM* creatine (pH=7.5), and incubated for 10 *min* at 37*°C*. The reaction was stopped by addition of 2 *ml* of 2% dimethyl aminobenzaldehyde in dimethyl sulfoxide and 15 *ml* HCl. After 20 *min* at 25*°C*, the optical density was measured at 435 *nm*. One unit of creatinase activity was defined as the amount of enzyme that liberated 1 *μmol* of *urea/min/ml* under standard conditions ^22^.

## Results

### Amplification of cre gene and construction of pET28a-cre plasmid

**Figure 1.**
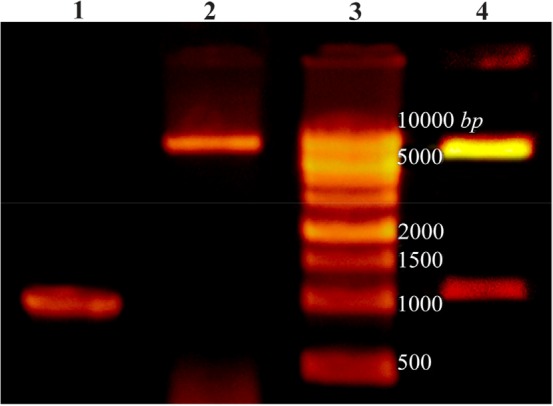
Lane 1: PCR product of Cre gene, lane 2: pET28a-cre plasmid extraction result, lane 3: DNA ladder, lane 4: double digestion of recombinant pET28a-cre by NheI and XhoI. Products were electrophoresed on 0.7% agarose gel.

PCR amplification of creatinase gene from *P. putida* ATCC 12633 genomic DNA led to a 1209 *bp* single band (Figure 1). The PCR product and pET28a plasmid were digested using NheI and XhoI restriction enzymes and ligated and transformed to chemical competent *DH5α E. coli* cells. Cloning of recombinant creatinase was confirmed by colony PCR, double digestion using NheI and XhoI and sequencing. Double digestion of recombinant plasmid (pET28a-cre) led to two bands of 5369 *bp* and 1209 *bp* according to linear pET28a and cre gene sizes, respectively (Figure 1).

### Expression and western blot analysis

**Figure 2.**
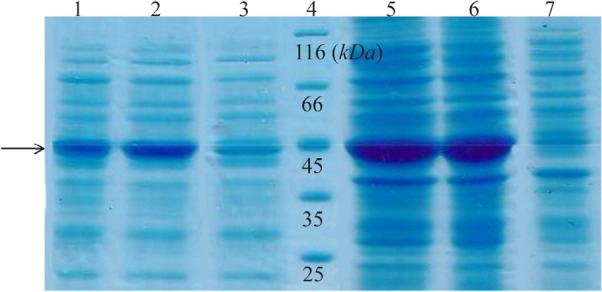
SDS-PAGE analysis of recombinant creatinase expression. Lane 1 and 2 are soluble fractions of induced *BL21* (containing pET28a-cre) for 4 and 16 *hr* and 3 is soluble fraction of induced negative control. Lane 4 is the protein ladder. Lane 5 and 6 are crude extract of total protein of induced *BL21* (containing pET28a-cre), and lane 7 is the negative control.

**Figure 3.**
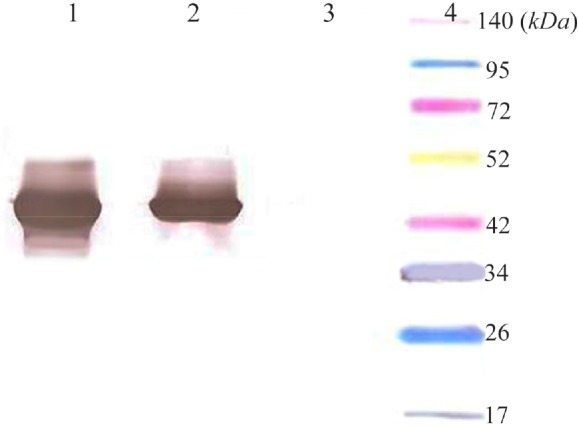
Western blot analysis of expressed creatinase using anti-His-tag monoclonal antibody conjugated to HRP. Lane 1: total protein, lane 2: soluble protein*,* lane 3: total protein in negative control, lane 4: thermo scientific Spectra Multicolor Broad Range Protein marker.

Total and soluble forms of recombinant enzyme were analyzed using SDS-PAGE (Figure 2). For increasing the amount of soluble protein and lysis of whole bacterial cells, different lysis methods were tested and grinding with liquid nitrogen was the best (data was not shown). Estimation of expression yield by AlphaEase software showed the creatinase was expressed in high yield (57% of the total protein) and the majority of the protein was in soluble form (Figure 2). To confirm the expression of desired protein, western blot analysis was performed using rabbit anti-His-tag monoclonal antibody conjugated to HRP. The single band with a molecular weight of approximately 45 *kDa* appeared (Figure 3).

### Purification of recombinant creatinase

**Figure 4.**
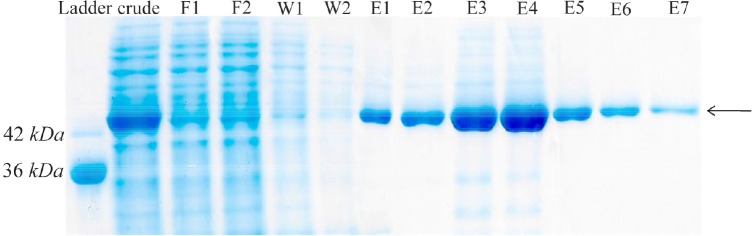
Protein purification with Ni-NTA agarose resin. F1–F2: different fractions of flow-through, W1–W2: different fractions of wash, E1–E7: different fractions of elution.

Finally, recombinant creatinase was purified under native condition using Ni-NTA affinity chromatography and fractions were observed in SDS-PAGE (Figure 4). Purity percent of different fractions was estimated by AlphaEase software and minimum purity percent of fractions was 98%.

### Plasmid stability

The results of plasmid stability test showed that the number of transformed colonies with pET28a-cre in plates containing antibiotics were over 50% of colonies in plates without antibiotics, thus this plasmid demonstrated good stability (data was not shown).

### Concentration and activity of recombinant enzyme

Based on the total protein concentration and percentage of the creatinase in total protein (estimated using AlphaEase software), the concentration of recombinant enzymes that was expressed in different temperatures of 22, 28 and 37°*C* were measured. The enzyme which was expressed at 28°*C* had the highest concentration (0.727 *mg/ml*) (Table 1). The enzyme activity of creatinase which was expressed in different induction temperatures (22, 28 and 37*°C*) was measured ^33^ and the results showed that the activity of enzyme that induced at 37*°C* was more than two other samples (Table 1).

**Table 1.** Concentration and specific activity of recombinant creatinase which expressed in different induced temperatures (22, 28 and 37*°C*)

**Samples**	**Absorbance at 595 *nm***	**Sample concentration (*mg/ml*)**	**Absorbance at 435 *nm***	**Activity (*unit/ml*)**	**Specific activity (*unit/mg*)**
**Recombinant creatinase expressed at 22°*C***	1.194	0.437	1.18	11.02	25.23
**Recombinant creatinase expressed at 28°*C***	1.848	0.727	1.42	13.27	18.53
**Recombinant creatinase expressed at 37°*C***	1.4	0.438	1.24	11.58	26.54

## Discussion

The muscular creatine and phosphocreatine are changed into creatinine nonenzymatically at a constant rate which disperses out the cell and is excreted into urine by kidney. Increasing the serum concentration and decreasing the renal clearance of creatinine are indicative of the progression of renal disease ^34^ and measuring creatinine concentration is much important in diagnosis of muscle, kidney and thyroid gland health and function. Several methods for the determination of creatinine in biological fluids have been described ^10^. Kepller *et al* compared a colorimetric picric acid, an enzymatic and a High Performance Liquid Chromatography (HPLC) method to assess their appropriateness for routine measurement of creatinine. The colorimetric jaffe method showed interferences with some substances in biological fluids. But comparison between HPLC and the enzymatic measurement gave a good agreement ^35^. In another study, it was shown that albumin, IgG and HbF interferences with jaffe reaction lead to large nonspecific variances and inaccurate GFR estimates especially in children and neonates, so enzymatic method is preferred and enzymatic method should become the method of choice for evaluating kidney function with samples from pediatric patients ^36^. On the other hand, in diabetic patients, blood sugar and acetone make interferences ^37,38^.

A large amount of creatinase enzyme is required for clinical use so in this study recombinant DNA technology was used to obtain an overproducing strain and a high creatinase production yield that high productivity and fusion tags lead to simplification of purification process and improvement of the quality of the enzyme.

**Figure 5.**
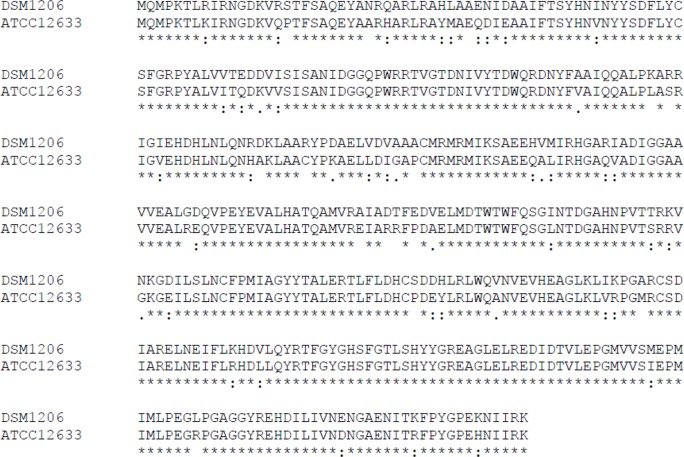
Alignment of *P. putida ATCC12633* creatinase with *P. putida DSM1206* creatinase.

Creatinase of *Flavobacterium sp*. (strain u-188) ^1^, *Bacillus sp*. (strain B-0618) ^11^ and *P. putida* DSM2106 (*Arthrobacter siderocapsulatus*) ^23^ have been completely recognized and well characterized and have been used in commercial kits. The highest specific activity of these native creatinases was about 9 *u/mg* protein. Hoeffken *et al* reported the activity of *P. putida* creatinase purified by various methods of purification from 4.03 to 8.9 *u/mg* ^23^. The specific activity of recombinant creatinase ^37,38^ expressed in *E. coli* JM109 has been 1.8 *u/ml* ^1^ and also Ming Chung Chang has reported recombinant creatinase activity at 0.04 *u/ml* ^26^ while the activity of our recombinant creatinase (*P. putida* PTCC1694) was about 26.54 *u/mg*, 3 fold more than the above mentioned creatinase. The multiple sequence alignment of *P. putida* ATCC-12633 and *P. putida* DSM1206 creatinases revealed a large degree of homology. The overall amino acid identity of these two enzymes was 85.30% (Figure 5).

As mentioned, high productivity yield is so important in producing clinical enzymes. Some studies indicated recombinant creatinase expressed as inclusion bodies; to circumvent this problem, they used signal peptide and produced creatinase as secretory protein to increase protein folding and solubility but 50% of total protein remains unprocessed containing signal peptide which has no activity ^2^. But in our recombinant expression system, the creatinase is completely soluble and 57% of total protein is the recombinant enzyme. So, in this study, *P. putida* ATCC-12633 was introduced as a new source of highly active creatinase for using in clinical application.

## Conclusion

The *P. putida ATCC12633* recombinant creatinase was expressed efficiently in *E. coli BL21* and 57% of total protein was the recombinant creatinase. On the other hand, the expressed creatinase has high solubility and also the enzyme has good activity compared to enzymes used in commercial kits, so a new source of creatinase for creatinine assay kit was produced.
